# A Guanosine‐Quadruplex Hydrogel as Cascade Reaction Container Consuming Endogenous Glucose for Infected Wound Treatment—A Study in Diabetic Mice

**DOI:** 10.1002/advs.202103485

**Published:** 2022-01-22

**Authors:** Yuanfeng Li, Linzhu Su, Yongxin Zhang, Yong Liu, Fan Huang, Yijin Ren, Yingli An, Linqi Shi, Henny C. van der Mei, Henk J. Busscher

**Affiliations:** ^1^ State Key Laboratory of Medicinal Chemical Biology Key Laboratory of Functional Polymer Materials Ministry of Education Institute of Polymer Chemistry College of Chemistry Nankai University 94 Weijin Road Tianjin 300071 P. R. China; ^2^ University of Groningen and University Medical Center Groningen Department of Biomedical Engineering Antonius Deusinglaan 1 Groningen 9713 AV The Netherlands; ^3^ Wenzhou Institute, University of Chinese Academy of Sciences Oujiang Laboratory (Zhejiang Lab for Regenerative Medicine, Vision and Brain Health) 1 Jinlian Road, Longwan District Wenzhou 325001 P. R. China; ^4^ Tianjin Key Laboratory of Radiation Medicine and Molecular Nuclear Medicine Institute of Radiation Medicine Chinese Academy of Medical Science & Peking Union Medical College 238 Baiti Road Tianjin 300192 P. R. China; ^5^ University of Groningen and University Medical Center Groningen Department of Orthodontics Hanzeplein 1 Groningen 9700 RB The Netherlands

**Keywords:** bacterial infection, cascade reactions, diabetic foot ulcers, reactive‐oxygen‐species, supramolecular hydrogels

## Abstract

Diabetic foot ulcers infected with antibiotic‐resistant bacteria form a severe complication of diabetes. Antimicrobial‐loaded hydrogels are used as a dressing for infected wounds, but the ongoing rise in the number of antimicrobial‐resistant infections necessitates new, nonantibiotic based designs. Here, a guanosine‐quadruplex (G_4_)‐hydrogel composed of guanosine, 2‐formylphenylboronic acid, and putrescine is designed and used as a cascade‐reaction container. The G_4_‐hydrogel is loaded with glucose‐oxidase and hemin. The first cascade‐reaction, initiated by glucose‐oxidase, transforms glucose and O_2_ into gluconic acid and H_2_O_2_. In vitro, this reaction is most influential on killing *Staphylococcus aureus* or *Pseudomonas aeruginosa* in suspension, but showed limited killing of bacteria in biofilm‐modes of growth. The second cascade‐reaction, however, transforming H_2_O_2_ into reactive‐oxygen‐species (ROS), also enhances killing of biofilm bacteria due to hemin penetration into biofilms and interaction with eDNA G‐quadruplexes in the biofilm matrix. Therewith, the second cascade‐reaction generates ROS close to the target bacteria, facilitating killing despite the short life‐time of ROS. Healing of infected wounds in diabetic mice proceeds faster upon coverage by these G_4_‐hydrogels than by clinically common ciprofloxacin irrigation. Moreover, local glucose concentrations around infected wounds decrease. Concluding, a G_4_‐hydrogel loaded with glucose‐oxidase and hemin is a good candidate for infected wound dressings, particularly in diabetic patients.

## Graphical abstract

A supramolecular guanosine‐quadruplex (G_4_)‐hydrogel containing glucose oxidase and hemin provides a cascade‐reaction container suitable as an antibacterial dressing for infected wounds. The cascade‐reaction in the G_4_‐hydrogel generates reactive‐oxygen‐species inside an infectious biofilm from endogenous glucose, killing multidrug resistant bacteria on a nonantibiotic basis, yielding faster healing of infected wounds, and reducing local glucose levels in diabetic mice.

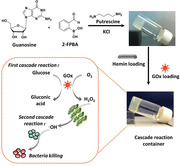

## 1 Introduction

New antimicrobials with nonantibiotic working mechanisms are direly needed to counter the threat of antimicrobial‐resistant infection becoming the number one cause of death in due time. Bacterial infection presents a major problem in burn wound patients and in patients with infected wounds also carrying other diseases. Diabetic foot ulcers for instance, are amongst the most severe complications of diabetes and are prevalent in over 25 million people worldwide. This number is estimated to double by 2030.^[^ ^1^, ^2^ ^]^ One fourth of all diabetic patients will finally have a limb amputation 6–18 months after being diagnosed with a diabetic foot ulcer.^[^ ^3^, ^4^ ^]^ Clinical treatment of diabetic foot ulcers includes debridement and drug administration in order to enhance revascularization.^[^ ^3^ ^]^ Treatment of infected diabetic foot ulcers is more difficult as many antimicrobials rapidly lose their efficacy due to an alarming increase in the number of antimicrobial‐resistant bacterial pathogens.^[^ ^5^ ^]^ Moreover, infection of diabetic foot ulcers is due to bacteria in a biofilm‐mode of growth in which they are well protected against conventional antimicrobial penetration and killing.^[^ ^6^, ^7^ ^]^

Wound dressings speed up healing of diabetic foot ulcers as they preserve a moist environment and are impermeable to bacteria which aids to prevent infection.^[^ ^3^, ^8^ ^]^ Hydrogels have several features in common with human soft tissue, including flexibility, high water content, and high capacity to absorb wound exudate and are therefore ideal as a wound dressing.^[^ ^9^ ^]^ Moreover, hydrogels can be loaded with antimicrobials for the treatment of infected wounds, including infected diabetic foot ulcers.^[^ ^8^, ^10^ ^]^

**Figure 1:**
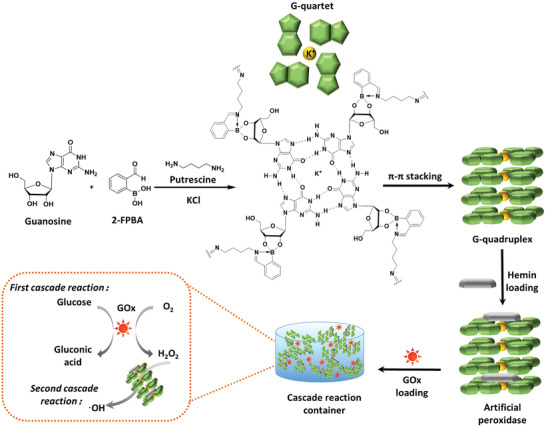
A mixture of guanosine (G), 2‐formylphenylboronic acid (2‐FPBA), and putrescine is reacted to form G‐quartets stabilized with K^+^. G‐quartets self‐assemble through *π*–*π* stacking to a G‐quadruplex structure forming a supramolecular, biomimetic G_4_‐hydrogel. The G_4_‐hydrogel is loaded with hemin and glucose oxidase (GOx). In the first cascade reaction, GOx catalyzes the transformation of glucose and O_2_ into H_2_O_2_, while the combination of the G‐quadruplex and hemin acts as an artificial peroxidase to produce hydroxyl radicals (•OH) from H_2_O_2_ in the second reaction of the cascade.

Here, a supramolecular guanosine‐quadruplex (G_4_)‐hydrogel was used as a cascade reaction container to produce reactive‐oxygen‐species (ROS) from glucose (**Figure** **1**). Cascade reactions integrate two or more reactions and each completed reaction initiates the next reaction.^[^ ^11^ ^]^ For use as a cascade reaction container, G_4_‐hydrogels were loaded with glucose‐oxidase and hemin, to transform naturally occurring endogenous glucose into H_2_O_2_ in the first, glucose‐oxidase assisted reaction. In the subsequent reaction, H_2_O_2_ is transformed into ROS with the aid of hemin. ROS exhibits multiple ways of antibacterial activity and can disrupt bacterial cell membranes, damage intracellular DNA, and disintegrate the extracellular polymeric substance matrix of infectious biofilms that forms the glue holding an infectious biofilm together.^[^ ^12^, ^13^, ^14^ ^]^ Use of cascade reactions for bacterial infection‐control has seldom been explored and importantly may kill infectious bacteria on a nonantibiotic basis. G_4_‐hydrogels loaded with cascade reaction components may be particularly suitable for the eradication of bacteria from infected diabetic foot ulcers, with the potential of decreasing endogenous glucose concentrations.

## 2 Results

### 2.1 Self‐Assembly of Guanosine‐Quadruplex Hydrogels and Loaded with Glucose‐Oxidase and Hemin

G_4_‐hydrogels could be self‐assembled from a mixture of guanosine, 2‐formylphenylboronic acid (2‐FPBA), putrescine (Put) and potassium chloride (KCl) at a molar ratio of 1:1:0.5:0.25 in ultrapure water (Figure S1a, Supporting Information). For self‐assembly, guanosine was first coupled with 2‐FPBA (Figure S2a, Supporting Information), as evidenced by the disappearance of boronic —B—OH peak observed in the ^11^B NMR spectrum of 2‐FPBA (*δ* = 30.26 ppm). Instead, a broad —B—OC— peak around *δ* = 0 ppm occurred (Figure S2b, Supporting Information). Concurrently in ^1^H NMR spectra, aldehyde —CHO peaks of 2‐FPBA (*δ* = 9.89 ppm) disappeared upon reaction with putrescine and imino —CH═N‐ peaks (*δ* = 8.36 ppm) appeared (Figure S2c, Supporting Information). Fourier transform infrared (FTIR) spectra also confirmed formation of iminoboronate bonds after gelation (Figure S3, Supporting Information). Coupling of guanosine with 2‐FPBA was furthermore supported by the disappearance of free O—H (3350–3067 cm^−1^) and B—OH (1296 cm^−1^) FTIR absorption bands (Figure S3, Supporting Information). Formation of iminoboronate bonds gave rise to the development of new B—OC (1011–1022 cm^−1^) FTIR absorption bands. Binding between putrescine and 2‐FPBA in hydrogels was supported by the disappearance of the C═O (1662 cm^−1^) and the appearance of the C═N (1696 cm^−1^) FTIR absorption band (see also Figure S3, Supporting Information).

Hemin loading was done by self‐assembled gelation of G_4_‐hydrogel in presence of hemin. Gelation could only be initiated when hemin concentrations were 0.36 g L^−1^ or less. At higher concentrations, a liquid solution remained (Figure S1b, Supporting Information). Self‐assembled gelation in presence of 0.36 g L^−1^ hemin was not affected by the additional presence of glucose oxidase (GOx) during loading, regardless of the concentration applied (Figure S1c, Supporting Information). Importantly, GOx/hemin loaded G_4_‐hydrogels remained stable up to at least 4 weeks, albeit with a mild discoloration (Figure S1d, Supporting Information). Accordingly, in the remainder of this article, all G_4_‐hydrogels were self‐assembled in presence of 0.36 g L^−1^ hemin to yield the maximal possible transformation of H_2_O_2_ into ROS.

Short term exposure (3 h) to this concentration of hemin, did not affect the viability of human skin fibroblasts as compared with exposure to phosphate buffer saline (PBS) (Figure S4a, Supporting Information). The viability of fibroblasts was more readily affected by H_2_O_2_ generation in the first cascade reaction during short term exposure to GOx loaded G_4_‐hydrogels (Figure S4b, Supporting Information), for which reason the GOx concentration during self‐assembly was confined to 0.25 g L^−1^ in the remainder of this article. Note, that the impact of short term exposure to G_4_‐hydrogels self‐assembled in presence of 0.25 g L^−1^ GOx and 0.36 g L^−1^ hemin (Figure S4c, Supporting Information) reduced fibroblast viability to 48%, but upon long term exposure (24 h) further reduction was observed to 33% (Figure S4d, Supporting Information). Long term exposure of unloaded human skin fibroblasts to unloaded G_4_‐hydrogels was not significantly different as upon exposure to PBS (see also Figure S4d, Supporting Information).

**Figure 2:**
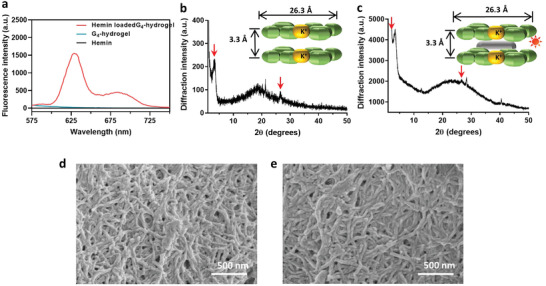
a) Fluorescence emission spectra of hemin, G_4_‐hydrogels, and hemin in G_4_‐hydrogels. b) X‐ray powder diffraction of lyophilized G_4_‐hydrogels, with characteristic Bragg angles indicated by arrows. c) X‐ray powder diffraction of lyophilized G_4_‐hydrogels after glucose‐oxidase/hemin loading, with characteristic Bragg angles indicated by arrows. d) Scanning electron micrographs of lyophilized G_4_‐hydrogels. e) Scanning electron micrographs of lyophilized G_4_‐hydrogels after glucose‐oxidase/hemin loading.

Fluorescence emission spectra demonstrated hemin binding with G‐quartets in G_4_‐hydrogels through *π*–*π* stacking yielding characteristic^[^ ^15^, ^16^ ^]^ fluorescence emission between 575 and 750 nm, that was absent in G_4_‐hydrogels without hemin or hemin in water (**Figure** **2**a). X‐ray powder diffraction (XRD) on lyophilized powder of G_4_‐hydrogels demonstrated two characteristic Bragg diffraction peaks at 2*θ* values 3.1° and 26.5°, regardless of GOx/hemin loading (compare Figure 2b,c). The smaller Bragg angles reflected the width of the square planar structure of the guanosine‐quartet (26.3 Å). The larger Bragg angles reflected the distance between two G‐quartets (3.3 Å) held together by K^+^ ions. Morphologically, lyophilized G_4_‐hydrogels demonstrated fibrous networks stacked as a layer‐by‐layer structure regardless of GOx/hemin loading (compare Figure 2d,e), similar as in other G‐quadruplex hydrogels.^[^ ^17^, ^18^, ^19^ ^]^

### 2.2 Verification of Cascade Catalytic Ability of Glucose Oxidase/Hemin Guanosine‐Quadruplex‐Hydrogels at Different Glucose Concentrations

**Figure 3:**
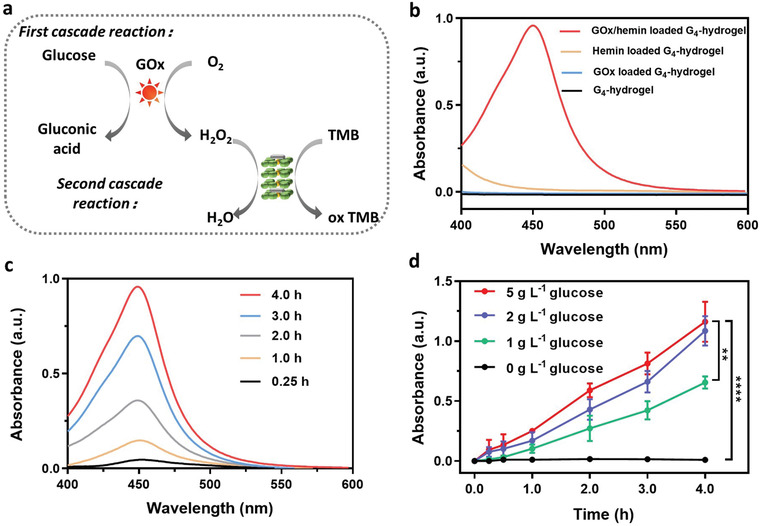
Demonstration of the occurrence of a cascade reaction initiated by exposure of a GOx/hemin‐loaded G_4_‐hydrogels based on TMB oxidation. a) Overview of the cascade reactions occurring. b) UV–vis absorption spectra of a TMB solution in the presence (4 h) of differently loaded G_4_‐hydrogels and glucose (2 g L^−1^). c) UV–vis absorption spectra of a TMB solution in presence of a GOx/hemin‐loaded G_4_‐hydrogel and glucose (2 g L^−1^) for 0–4 h. d) UV–vis absorption at 450 nm of a TMB solution in presence of a GOx/hemin‐loaded G_4_‐hydrogel at different glucose concentrations as a function of time. All data in panel (d) were expressed as means ± standard deviations over triplicate experiments with separately prepared hydrogels. Asterisks indicate statistical significance at **p < 0.01 and ****p < 0.0001 (one‐way ANOVA test) between differences with respect to exposure to the hydrogel with the highest glucose loading (5 g L^−1^).

In order to verify whether the proposed cascade reaction (see Figure 1) indeed occurred in G_4_‐hydrogels loaded with GOx and hemin, chromogenic 3,3′,5,5′‐tetramethylbenzidine (TMB) was used as a peroxidase substrate. TMB can be oxidized to ox‐TMB by enzymes with peroxidase‐like activity in the presence of H_2_O_2_ (**Figure** **3**a).^[^ ^20^, ^21^ ^]^ After 4 h in PBS (pH 7.4) containing TMB (5 mm) and supplemented with 2 g L^−1^ glucose, only G_4_‐hydrogels loaded with GOx/hemin demonstrated an increase in absorbance at 450 nm, but unloaded hydrogels or hydrogels loaded with only hemin or GOx did not yield an increase of UV–vis absorbance at 450 nm (Figure 3b). The absorbance at 450 nm increased over time, indicating an increase in the concentration of hydroxyl radicals (Figure 3c). Absorbances increased significantly with increasing glucose concentrations up till ≈2 g L^−1^ and hardly yielded any further increase in absorbance (Figure 3d). These experiments show that the cascade reactions envisaged in Figure 1 occur successfully upon exposure to environmental glucose. Further in vitro experiments were carried out at glucose concentrations between 2 to maximally 5 g L^−1^, as higher glucose concentrations yielded no further increase in cascade reaction output. Note, these levels exceed healthy glucose levels (1.4 g L^−1^) and are indicative of diabetes (>2.0 g L^−1^).^[^ ^22^ ^]^

### 2.3 Delineation of Bacterial Killing by Different Reactions in the Cascade

In order to delineate the effects of each of the two reactions in the cascade occurring in our G_4_‐hydrogel containers, Gram‐positive *Staphylococcus aureus* (*S. aureus*) Xen36 and Gram‐negative *Pseudomonas aeruginosa* (*P. aeruginosa*) Xen41 were grown planktonically and in a biofilm‐mode of growth in presence with G_4_‐hydrogels loaded with 0.25 g L^−1^ GOx and/or 0.36 g L^−1^ hemin. For these experiments, growth media were supplemented with 5 g L^−1^ glucose.

**Figure 4:**
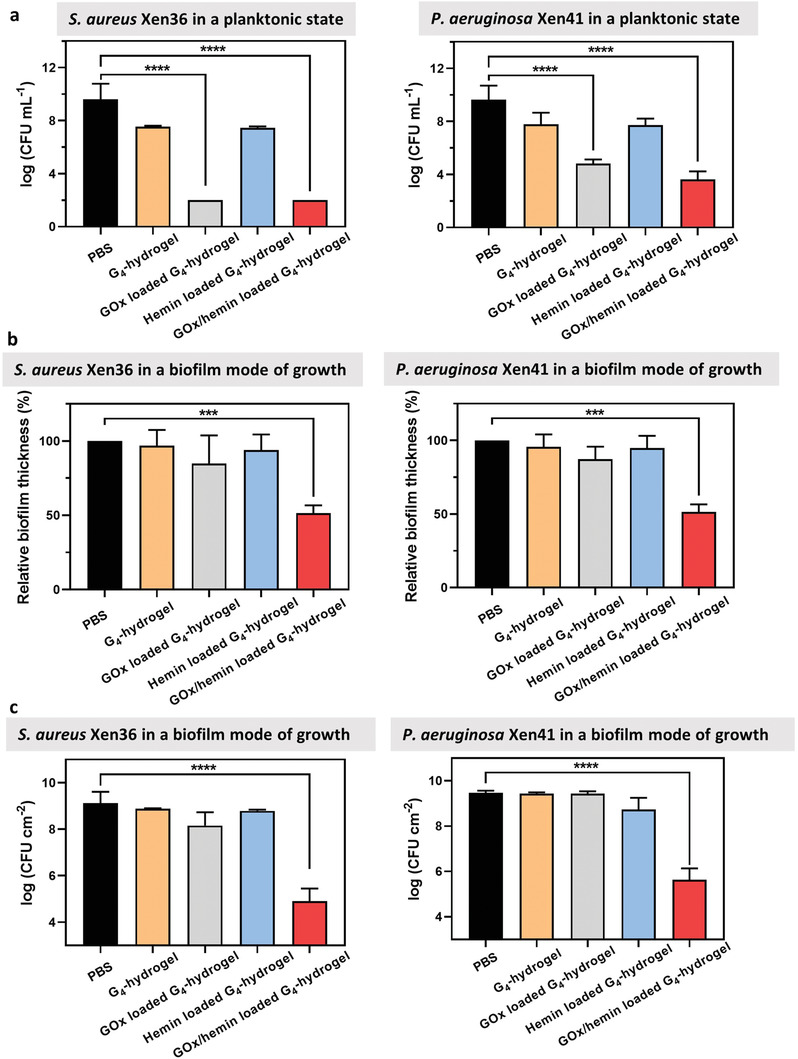
Delineation of bacterial killing by different reactions in the cascade in tryptone soy broth supplemented with glucose (5 g L^−1^). a) The number of planktonic *S. aureus* Xen36 or *P. aeruginosa* Xen41 in suspension after 3 h growth in presence of differently loaded G_4_‐hydrogels. Data were expressed as means ± standard deviations over triplicate experiments with independently grown biofilms and separately prepared hydrogels. b) Relative thickness of *S. aureus* Xen36 or *P. aeruginosa* Xen41 biofilms after exposure to differently loaded G_4_‐hydrogels for 8 h, setting the biofilm thickness in PBS in absence of hydrogel exposure at 100%. Data were expressed as means ± standard deviations over triplicate experiments with independently grown biofilms and separately prepared hydrogels. Biofilm thicknesses were measured on five randomly chosen spots on each biofilm. c) The number of CFU cm^−2^ after exposure of *S. aureus* Xen36 or *P. aeruginosa* Xen41 biofilms to differently loaded G_4_‐hydrogels for 8 h. Data represent means ± standard deviations over triplicate runs with different cultures and separately prepared hydrogels. Asterisks indicate statistical significance at ***p < 0.001 and ****p < 0.0001 (one‐way ANOVA test) between differences with respect to exposure to PBS in absence of hydrogels.

Neither PBS nor unloaded G_4_‐hydrogels or loaded with only hemin yielded any killing of planktonic bacteria in suspension (**Figure** **4**a), regardless of their Gram‐character. Loading of G_4_‐hydrogels with only GOx yielded significant decreases in CFUs for both strains, attributed to the generation of hydrogen peroxide. Loading with both GOx and hemin yielded only a slightly faster killing (see Figure S5, Supporting Information, for kinetics), but at end‐point (Figure 4a) synergistic effects of loading with both GOx and hemin were absent in killing of planktonic bacteria. Likely, the short life‐time of ROS is insufficient for the ROS to diffuse from its generation site within the G_4_‐hydrogel to planktonic bacteria in suspension.

**Figure 5:**
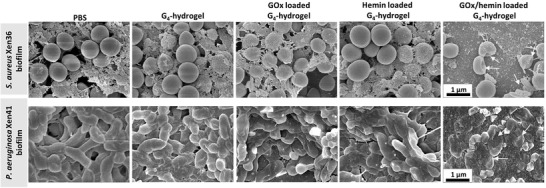
Scanning electron micrographs of *S. aureus* and *P. aeruginosa* in a biofilm‐mode of growth in contact with unloaded G_4_‐hydrogels or hydrogels loaded with only GOx or hemin or GOx/hemin in tryptone soy broth supplemented with glucose (5 g L^−1^). Biofilms were 48 h old and contacted with G_4_‐hydrogels for 8 h. Arrows point to cell wall damaged bacteria.

Delineation of both cascade reactions toward bacterial killing in a biofilm‐mode of growth yielded a different conclusion. When the G_4_‐hydrogels were evaluated toward bacteria in their biofilm‐mode of growth, neither PBS nor unloaded G_4_‐hydrogels or hydrogels loaded with only GOx or only hemin yielded any significant reductions in biofilm thickness or numbers of CFUs in biofilms of *S. aureus* Xen36 or *P. aeruginosa* Xen41 (Figure 4b,c). Reductions in biofilm thickness measured after fluorescence staining using confocal laser scanning microscopy (CLSM) were confirmed by biomass measurements on crystal violet stained biofilms (Figure S6, Supporting Information). Killing of bacteria in a biofilm‐mode of growth was solely observed using G_4_‐hydrogels loaded with GOx and hemin, that is, by means of the second reaction in the cascade. Both *S. aureus* as well as *P. aeruginosa* in biofilms exposed to G_4_‐hydrogels loaded with GOx and hemin had microscopically observable cell wall damage (**Figure** **5**) that was absent after biofilm exposure to unloaded or G_4_‐hydrogels loaded only with GOx or hemin. This suggests that the second cascade reaction generating ROS occurs inside a bacteria, in the close vicinity of the target bacteria.

**Figure 6:**
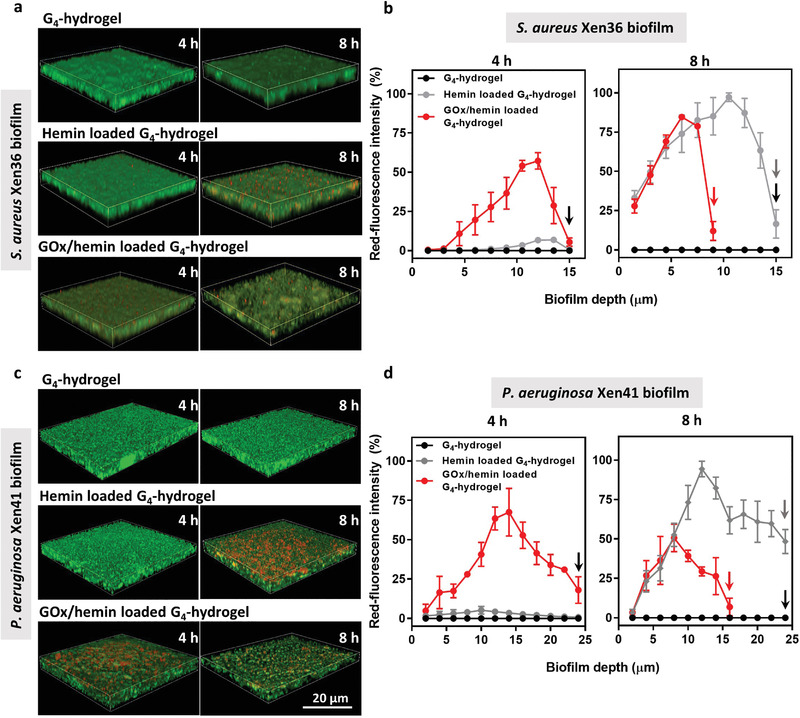
Hemin penetration and accumulation in 48 h old *S. aureus* Xen36 and *P. aeruginosa* Xen41 biofilms grown upon exposure (4 or 8 h) to unloaded, hemin, or GOx/hemin‐loaded G_4_‐hydrogels in tryptone soy broth supplemented with glucose (5 g L^−1^). Note the thicknesses of biofilms exposed upon 4 or 8 h exposure differed, as indicated by the arrows in each graph. a) Merged CLSM images of green‐fluorescent *S. aureus* biofilms showing red‐fluorescent hemin penetration after 4 and 8 h of growth in contact with the hydrogel. CLSM images were taken on five randomly chosen spots on each biofilm. b) Red‐fluorescence intensity due to hemin penetration in *S. aureus* biofilms as a function of biofilm depth, normalized with respect to the highest fluorescence intensity observed after 8 h of growth, set at 100%. Depth is measured from the surface of the biofilm in contact with the hydrogel to the bottom of the confocal dish. c) Same as panel (a), now for *P. aeruginosa* biofilms. d) Same as panel (b), now for *P. aeruginosa* biofilms. All data in panels (b) and (d) were expressed as means ± standard deviations over triplicate experiments with independently grown biofilms and separately prepared hydrogels.

In order to verify this suggestion, we evaluated hemin penetration and accumulation into the biofilms from hemin and GOx/hemin loaded G_4_‐hydrogels into *S. aureus* (**Figure** **6**a,b) and *P. aeruginosa* (Figure 6c,d) biofilms. Quantitative analysis of red‐fluorescence intensities due to hemin penetration and accumulation in biofilms (Figure 6a,c), indicated that hemin accumulated in biofilms after 8 h of exposure to G_4_‐hydrogels (Figure 6b,d). However, after 4 h of exposure to hydrogels, hemin was only found accumulated in biofilms in contact with GOx/hemin loaded G_4_‐hydrogels and not after contact with G_4_‐hydrogels loaded with only hemin (Figure 6b,d). Thus, it is concluded that the acidic conditions created in the first GOx‐initiated cascade reaction caused favorable conditions for hemin dissociation from G‐quartets in G_4_‐hydrogels allowing their penetration into the biofilms. Accumulation of hemin in biofilm matrix is subsequently ensured by hemin binding with recently described G‐quartets in the eDNA structure of the biofilm matrices.^[^ ^23^ ^]^ To further substantiate this, eDNA quartets in biofilms were visualized by binding of anti‐DNA G‐quadruplex antibodies and green‐fluorescently labeled goat anti‐mouse IgG, after exposure to a hemin‐loaded biofilm. CLSM images showed green‐fluorescent eDNA quadruplexes and red‐fluorescent hemin accumulated inside a biofilm. Pearson's correlation analysis of the positions of green‐ and red‐fluorescent pixels yielded correlation coefficients 0.47 or 0.34 for *S. aureus* or *P. aeruginosa* biofilms, respectively, indicating 47% or 34% of the hemin that had accumulated in the biofilm colocalized with eDNA quadruplexes. (Figure S7, Supporting Information). Thus, the first cascade reaction not only generates H_2_O_2_, but also stimulates release of hemin from G‐quartets in a G_4_‐hydrogel to stimulate their binding to eDNA quartets in the biofilm matrix to ensure ROS generation through the second cascade reaction close to the target bacteria.

Bacterial killing by GOx/hemin loaded G_4_‐hydrogels was maintained after 4 weeks storage of the hydrogels (Figure S8, Supporting Information), despite a discoloration observed (Figure S1d, Supporting Information), indicating that their effective shelf life is at least 4 weeks.

### 2.4 Eradication of a Staphylococcal Biofilm from an Infected Wound in Diabetic Mice

**Figure 7:**
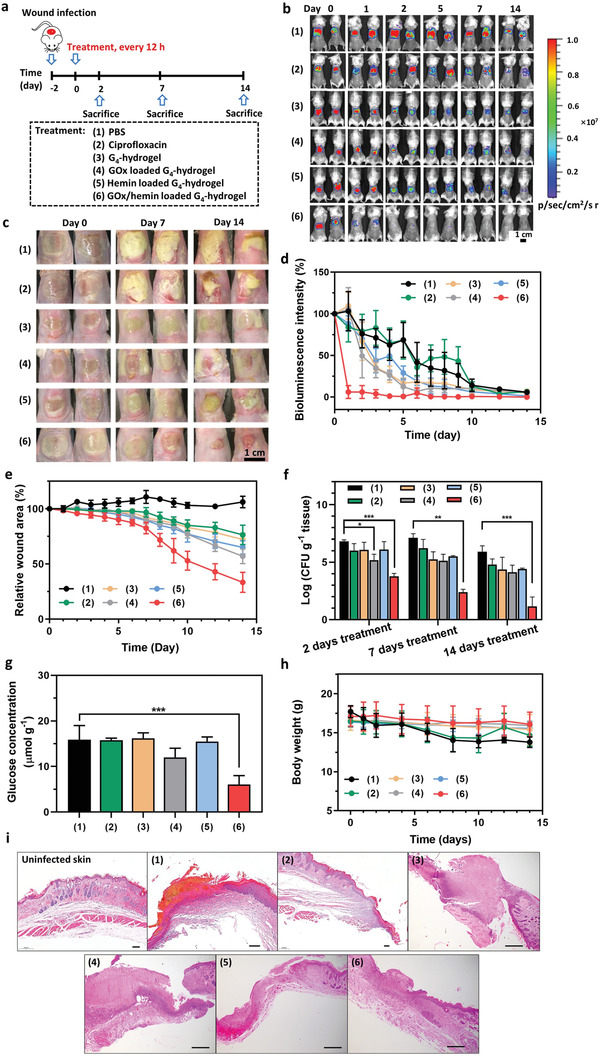
Eradication of staphylococcal biofilms in diabetic mice. a) Experimental scheme: *S. aureus* Xen36 was added onto open wounds (1 × 10^9^ bacteria per wound site) 2 days prior to initiating treatment. Treatment involved irrigation of the wound with PBS (100 µL) or a ciprofloxacin solution (100 µL, 2 µg mL^−1^) or coverage of the wound with 100 µL of differently loaded G_4_‐hydrogel. Irrigation was repeated and hydrogels replaced by fresh ones every 12 h during the entire experimental period till sacrifice. Note that sacrifice at days 2 and 7 was for evaluating the numbers of CFUs and blood analysis, while sacrifice at day 14 was done for the purpose of evaluating the numbers of CFUs, histology, and blood analysis. b) Bioluminescence images of differently treated mice at different times after initiating treatment in the same mouse. Two mice were randomly chosen as representatives of each group. c) Optical images of the wound area of differently treated mice at different times after initiating treatment in the same mouse. Two mice were randomly chosen as representatives of each group. d) Percentage bioluminescence intensity arising from the infection site of differently treated mice as a function of time after initiating treatment. Bioluminescence intensity at day 0 was set at 100%. Data are presented as means ± standard deviations over five mice in each group. e) Wound area of differently treated mice as a function of time after initiating treatment. Wound area was defined from optical images by taking the area representing unclosed, reddish inflamed skin or wound area covered with white excretion. Wound area at day 0 was set at 100%. Data are presented as means ± standard deviations over five mice in each group. f) CFUs per gram excised wound tissue at sacrifice of differently treated mice at days 2, 7, and 14 after initiating treatment. Data are presented as means ± standard deviations over three mice in each group. g) Local glucose concentrations in tissue surrounding an infected wound site, measured 4 h after initiating a treatment. Data are presented as means ± standard deviations over three mice in each group. h) Body weight of differently treated mice as a function of time after initiating treatment. Data are presented as means ± standard deviations over five mice in each group. i) Micrographs of hematoxylin/eosin stained, excised wound tissue of differently treated mice taken at sacrifice (day 14 after initiating treatment). Wound tissue was excised from the center of each wound. The scale bar equals 100 µm. Asterisks in all panels indicate statistical significances at * *p* < 0.05, **p < 0.01 and ***p < 0.001 (one‐way ANOVA) of differences with respect to PBS irrigation.

In order to evaluate the efficacy of GOx/hemin loaded G_4_‐hydrogels in eradicating staphylococci from infected wounds in diabetic mice, a wound was created on the dorsum of the mice (12 mm diameter) and inoculated with *S. aureus* Xen36. A dose finding pilot was conducted to determine the inoculation dose that yielded an infection that could be monitored over time using bioluminescence imaging. This pilot yielded the decision to use an inoculation dose of 1 × 10^9^ bacteria for each infected wound (Figure S9, Supporting Information). Subsequently, six groups of diabetic mice were created with thus infected wounds. Treatment started 2 days after inducing infection by irrigation with PBS, ciprofloxacin, or coverage of the infected wounds with differently loaded G_4_‐hydrogels (see **Figure** **7**a for experimental scheme). Ciprofloxacin was chosen for comparison of its efficacy with GOx/hemin‐loaded hydrogels as it is a frequently used, common, clinically applied antibiotic. Importantly, the minimal inhibitory and minimal bactericidal concentrations of *S. aureus* Xen36 against ciprofloxacin (and of four other common antibiotics) was similar as of clinical isolates from patients with diabetic foot ulcers (Table S1, Supporting Information).

Wound infection became evident from bioluminescent images (Figure 7b) as well as from optical imaging (Figure 7c). Moreover, bioluminescence as well as optical imaging showed superior and extremely fast eradication of the infection upon coverage with a G_4_‐hydrogel loaded with GOx/hemin as compared with PBS and ciprofloxacin irrigation, or coverage with otherwise loaded G_4_‐hydrogels. This conclusion was confirmed by analysis of the bioluminescence intensity (Figure 7d) and wound area (Figure 7e) over time as well as by CFU enumeration of viable staphylococci in excised wound tissue at the different times of sacrifice (Figure 7f). Concurrently, local glucose concentrations in wounds covered with GOx/hemin‐loaded G_4_‐hydrogels were significantly lower than when irrigated with PBS and ciprofloxacin irrigation, or coverage with otherwise loaded G_4_‐hydrogels (Figure 7g), indicating consumption of endogenous glucose in the first cascade reaction. Diabetic mice with their infected wounds covered with a G_4_‐hydrogel loaded with GOx/hemin suffered no measurable weight loss during the experimental period (Figure 7h), opposite to mice treated with PBS or ciprofloxacin irrigation. Histological analysis of wound tissue excised at sacrifice (day 14 after initiating treatment) demonstrated thickened epidermis and infiltration of immune cells upon treatment with PBS and ciprofloxacin, or coverage with unloaded G_4_‐hydrogels or hydrogels loaded with GOx or hemin only (Figure 7i). In GOx/hemin‐loaded G_4_‐hydrogel treated group, clear return to intact skin microstructures and re‐epithelialized epidermis was observed (Figure 7i). Routine blood parameters of all three groups were similar over the entire experimental period (Figure S10, Supporting Information).

## 3 Discussion

A new, supramolecular G_4_‐hydrogel loaded with GOx and hemin was designed and demonstrated to be suitable for use as an infected wound cover in diabetic mice, yielding extremely fast eradication of infection as compared with antibiotic irrigation of the wound and reduction of glucose concentration around the wound site. The G_4_‐hydrogel served as a cascade reaction container, in which glucose was enzymatically oxidized into H_2_O_2_ in a first reaction and H_2_O_2_ was subsequently transformed into ROS with the aid of the G‐quadruplex and hemin. Hemin was used for its *π*–*π* stacking ability with G‐quartets, resulting in a G‐quadruplex/hemin complex as a peroxidase‐mimicry.^[^ ^24^, ^25^ ^]^ The efficacy of the cascade reaction for killing of bacteria in an infectious biofilm depended to a major extend on the second cascade reaction, generating ROS inside a biofilm due to the ability of hemin to penetrate and accumulate into a biofilm. Although short term in vitro exposure of cell layers to G_4_‐hydrogels loaded with GOx and hemin indicated loss of viability, further loss of viability upon longer exposure (24 h) increased only slowly. Loss of viability upon long‐term exposure to G_4_‐hydrogels loaded with GOx and hemin was limited to 33%, which is at the threshold suggested to be critical for the survival of cellular layers under bacterial attack in vitro.^[^ ^26^ ^]^ The current evaluation of wound healing in vivo, shows wound healing was indeed not negatively impacted by exposure to G_4_‐hydrogels loaded with GOx and hemin. This confirms that loss of viability under in vitro conditions at the critical threshold of 33% does not necessarily have a negative impact on cellular survival under the more dynamic in vivo conditions.

Stable gelation of G_4_‐hydrogels was driven by three interactions: 1) Hoogsteen‐type hydrogen bonds between four guanine groups were formed in presence of potassium cations to generate planar G‐quartets;^[^ ^27^ ^]^ 2) iminoboronate bonds formed between diols in guanosine with primary amino groups in putrescine and aldehyde and boronic acid groups in 2‐FPBA, which connected G‐quartets together;^[^ ^28^ ^]^ 3) *π*–*π* stacking between G‐quartets to form a G_4_‐hydrogel in which water can be immobilized.^[^ ^19^ ^]^ G‐quadruplex hydrogels were first observed in cells, protecting the end‐caps of chromosomes.^[^ ^29^ ^]^ G‐quadruplex hydrogels therefore offer new opportunities for the preparation of biomimetic G_4_‐hydrogels and their use as a drug‐delivery device.^[^ ^30^ ^]^ Use of these biomimetic hydrogels as a container for cascade reactions by loading it with GOx and hemin, allowed to generate H_2_O_2_ from glucose and to generate ROS in a second cascade reaction to form ROS.

**Figure 8:**
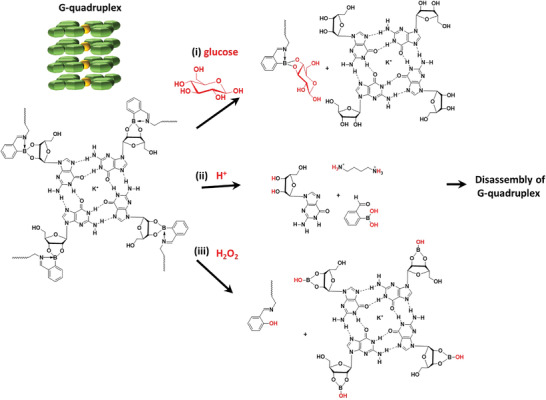
The disassembly mechanism of G‐quadruplex structures by i) glucose substitution of existing boronate ester bonds, breaking the connection between G‐quartets, ii) breaking hydrogen and iminoboronate bonds in G‐quartets through gluconic acid generation in the first cascade reaction, and iii) H_2_O_2_ oxidation of boronate ester bonds to phenol.^[^ ^28^, ^31^ ^]^

Whereas the second, hemin‐driven cascade reaction was of minor importance in the killing of planktonic bacteria suspended in an aqueous phase, it appeared crucial for killing bacteria in a biofilm‐mode of growth. This was attributed to the ability of hemin to penetrate and accumulate in biofilms through binding with eDNA G‐quadruplexes, facilitating generation of ROS in the close vicinity of their target bacteria in a biofilm. Moreover, hemin penetration was faster in the presence of hemin loaded G_4_‐hydrogels also loaded with GOx than in absence of GOx loading. Likely, *cis*‐diols in environmental glucose substitute guanosine form new cyclic boronate ester that break the connection between G‐quartets formed by existing boronate esters. At the same time, the first GOx driven cascade reaction yields gluconic acid and H_2_O_2_ that induce disassembly of the G‐quadruplex structure. Thus G‐quadruplex disassembly occurs by a variety of factors (**Figure** **8**) to stimulate hemin dissociation and release (see Figure S11, Supporting Information). Hemin subsequently diffuses into a biofilm together with the H_2_O_2_ produced to facilitate occurrence of the second cascade reaction inside the biofilm, close to its bacterial inhabitants to allow their killing despite the short live‐time of ROS.

Different Gram‐positive and Gram‐negative bacterial species have been identified in infected diabetic foot ulcers.^[^ ^32^ ^]^ Commonly, infected diabetic foot ulcers are locally treated with antibiotics or silver cations.^[^ ^33^, ^34^, ^35^, ^36^ ^]^ However, antibiotic efficacy is limited by the ongoing development of antibiotic resistance amongst bacterial pathogens^[^ ^37^, ^38^ ^]^ while bacterial resistance against silver cations has been reported due to genetic modification and horizontal gene transfer.^[^ ^39^, ^40^, ^41^ ^]^ ROS‐based eradication of infecting pathogens as resulting from GOx/hemin loaded G_4_‐hydrogels indiscriminately kills Gram‐positive and Gram‐negative bacterial pathogens^[^ ^42^ ^]^ on a nonantibiotic basis and accordingly also kills antibiotic‐resistant strains. In vivo efficacy was demonstrated in diabetic mice, as diabetic patients are likely to benefit most from our hydrogels due to their high endogenous glucose levels,^[^ ^22^ ^]^ but in vitro experiments also demonstrated good efficacy for glucose concentrations around 1 g L^−1^ representing nondiabetic conditions and suggesting clinical applicability of our G_4_‐hydrogels also for other bacterial skin infections.

## 4 Conclusions

A new, antibacterial, nonantibiotic containing wound cover was designed based on supramolecular G_4_‐hydrogels based on putrescine as a polyamine. The hydrogel acted as a cascade reactor when loaded with GOx and hemin. In the first cascade reaction, endogenous glucose was oxidized by GOx into H_2_O_2_, while in the second cascade reaction H_2_O_2_ was transformed into ROS with the aid of hemin bound to G‐quadruplexes. As a result, covering of infected open wounds in diabetic mice with these G_4_‐hydrogels yielded extremely fast eradication of infection as compared with antibiotic irrigation of the wound, concurrent with a reduction of the glucose concentration around an infected wound. Their ease of fabrication, long shelf‐life and self‐healing properties make G_4_‐hydrogels loaded with GOx and hemin a good candidate for antibacterial wound dressing for the treatment of infected diabetic foot ulcers. Prospects for the clinical translation of our biomimetic G‐quadruplex hydrogels as an antibacterial wound coverage are good, particularly because of their long shelf‐life, excellent tissue compatibility, and absence of negative tissue and blood reactions in mice. Use of endogenous glucose makes our G_4_‐hydrogels especially attractive for the treatment of infected diabetic foot ulcers. Therewith, prospects for the clinical translation of our biomimetic G‐quadruplex hydrogels as an antibacterial wound dressing are good.

## 5 Experimental Section

### Materials

Guanosine (G, 98%) and 2‐FPBA (97%) were purchased from Heowns Biochem Technologies (Tianjin, China). KCl (99.5%), glucose (99%), and hemin (98%) were purchased from J&K Scientific (Beijing, China). Putrescine (97%) was purchased from Sigma‐Aldrich (Shanghai, China). All reagents were used as received and aqueous solutions were prepared with ultrapure water (>18 MΩ) from a Millipore Milli‐Q system.

### Synthesis of Guanosine‐Quadruplex Hydrogels and Loading with Glucose‐Oxidase and Hemin

For the synthesis of G_4_‐hydrogels, guanosine (9.91 mg, 35 mmol), 2‐FPBA (5.25 mg, 35 mmol), putrescine (1.5 mg, 17.5 mmol), and KCl (0.66 mg, 8.75 mmol), were mixed in 1 mL ultrapure water and boiled until a clear solution resulted.

For preparing hemin loaded G_4_‐hydrogels, different amounts of hemin (0.36–0.54 mg) were dissolved in 0.2 m KOH and added into the boiling solution in preparing G_4_‐hydrogel. For preparing GOx/hemin loaded G_4_‐hydrogels, different amounts of GOx (0.125–0.5 mg) were added after cooling the solution in preparing hemin loaded G_4_‐hydrogel to 40 °C. The hydrogel was formed after the solution was gradually cooled down to room temperature.

### Characterization of Guanosine‐Quadruplex Hydrogels

For XRD, hydrogels were lyophilized and data were recorded on a D/Max‐2500 X‐ray diffractometer (Rigaku, Tokyo, Japan) with Cu K*α* radiation. FTIR spectra of lyophilized hydrogels in KBr tablet (1:100) were collected on an FTS 6000 FTIR instrument (Bio‐Rad, Hercules, California, America) using 128 scans at 8 cm^−1^ resolution. NMR spectra of the hydrogels were taken at 25 °C in deuterated water (D_2_O) using a Bruker Avance III 400 MHz spectrometer (for ^1^H NMR spectra) and an Ascend 400 MHz spectrometer (for ^11^B NMR spectra) (both were from Bruker, Fällanden, Switzerland). Binding of hemin to G‐quartets in G_4_‐hydrogels was demonstrated using a Hitachi F‐4600 fluorescence spectrophotometer (Tokyo, Japan) at 410 nm excitation and emission collection between 575 and 750 nm. For SEM, lyophilized hydrogels were put on conductive tape and sputter‐coated with gold for imaging. SEM images were taken on a JSM‐7500F field emission microscope (JEOL Ltd, Tokyo, Japan).

### Cascade Catalytic Ability of Guanosine‐Quadruplex Hydrogels Containing Glucose‐Oxidase and Hemin

In order to demonstrate the cascade catalytic ability of the G_4_‐hydrogels, hydrogels were prepared in tubes overnight and 2 mL of solution composed of glucose and 5 mm TMB in PBS (pH 7.4) was added on top of the hydrogels. Glucose concentrations were varied over the range of 1–5 g L^−1^, representing concentrations found in healthy (up to 2 g L^−1^) and diabetic patients (2–5 g L^−1^).^[^ ^22^ ^]^ After different time intervals up to 4 h, aliquots were taken and mixed with 0.5 mL 2 m H_2_SO_4_ to terminate the catalytic process. Hydroxyl radicals produced by the cascade reaction yielded oxidation of TMB that was monitored using UV–vis spectrophotometry (UV‐2550, Shimadzu, Kyoto, Japan) in the absorbance range between 400 and 600 nm. The experiments were done in triplicate with separately prepared hydrogels.

### Bacterial Strains, Growth Conditions, and Harvesting

Two multidrug‐resistant, bioluminescent strains were employed in this study: A Gram‐positive *S. aureus* Xen36 and a Gram‐negative *P. aeruginosa* Xen41 (PerkinElmer Inc., Waltham, MA, USA). Bioluminescent strains were facile for research purposes and the strains employed possessed an antibiotic‐resistance profile comparable with the profile of two clinical isolates from infected diabetic foot ulcers (*S. aureus* df1 and *P. aeruginosa* df2, see Table S1, Supporting Information).^[^ ^6^ ^]^ All strains were cultured at 37 °C in ambient air in tryptone soy broth (TSB). *S. aureus* Xen36 was cultured on TSB agar plates with 200 µg mL^−1^ kanamycin, and *P. aeruginosa* with 60 µg mL^−1^ tetracycline to preserve bioluminescence during culturing. One colony was inoculated in 10 mL TSB and incubated for 24 h at 37 °C and used to inoculate (1:20) 200 mL main culture and grown for 16 h. Bacterial cultures were harvested by centrifugation for 5 min at 5000 × *g*, washed twice with PBS (5 mm K_2_HPO_4_, 5 mm KH_2_PO_4_, and 150 mm NaCl, pH 7.0), and sonicated three times for 10 s (Vibra cell model 375, Sonics and Material Inc., Danbury, CT), while cooling in an ice/water bath to break possible aggregates. Finally, harvested bacteria were suspended in PBS to concentrations required in the respective experiments, as determined in a Bürker‐Türk counting chamber.

### Antibacterial Efficacy of Guanosine‐Quadruplex Hydrogels Containing Glucose‐Oxidase and Hemin

In order to study antibacterial efficacy of different hydrogels toward planktonic bacteria and delineate the antibacterial effects of both cascade reactions, different hydrogels were prepared overnight in 12‐wells plates. 1 mL of a bacterial suspension TSB (1 × 10^8^ bacteria per mL) with the addition of different amounts of glucose was put on top the hydrogels. After incubation for different time intervals up to 3 h at 37 °C, 10 µL aliquots were taken, serially diluted and plated on TSB agar, and incubated for 24 h at 37 °C after which the number of CFUs formed were counted.

In order to study antibacterial efficacy against bacteria in a biofilm mode of growth, 1 mL of a bacterial suspension in PBS suspension (10^8^ bacteria per mL) was added to confocal dishes (14 mm diameter) at 37 °C for 1 h to allow bacteria to adhere. Next, bacterial suspensions were removed and wells were washed with 1 mL PBS. 2 mL TSB was added into each well for 48 h at 37 °C for biofilm growth. The biofilms grown were covered with 500 µL hydrogel and 1500 µL TSB supplemented with 5 g L^−1^ glucose. Next, biofilms were scraped off the bottom of a dish and suspended in PBS by sonication for CFU enumeration (see above). Biofilm thickness was measured after staining with acridine orange using 3D CLSM (excitation 488 nm, fluorescence emission between 500 and 535 nm). Biofilm thicknesses were measured on five randomly chosen spots on each biofilm. In addition, biomass measurements were done using crystal violet staining (see Figure S6, Supporting Information).

### Hemin Penetration in Biofilms and Binding to Guanosine‐Quartets in eDNA

In order to study the penetration and accumulation of hemin in biofilms, 48 h biofilms were covered with G_4_‐hydrogels, hemin loaded G_4_‐hydrogel or GOx/hemin loaded G_4_‐hydrogels. After 4 or 8 h time intervals, biofilms were stained with acridine orange, followed by CLSM imaging excitation at 488 and 410 nm to excite stained bacteria and hemin, respectively, and measuring fluorescence between 500 and 535 nm (bacteria) and 583 and 680 nm (hemin).

In order to study the hemin binding to G‐quartets in eDNA present in the biofilm matrix, 48 h biofilms were covered with GOx/hemin loaded G_4_‐hydrogels for 4 h. Next, biofilms were fixed in 4% paraformaldehyde for 15 min at room temperature, washed three times with PBS and exposed to 5% fetal bovine serum for 1 h, followed by treatment of G‐quadruplex DNA specific primary antibody (Sigma‐Aldrich, CAT#MABE1126) for 1 h, and washed three times with PBS. Subsequently, green‐fluorescently labeled (Alexa Fluor 488) goat anti‐mouse IgG (Solarbio, CAT#SF131) was added for 1 h, followed by three times washing with PBS. eDNA G‐quadruplex nucleic acid structures and penetration of hemin in the biofilms were visualized using CLSM.^[^ ^23^ ^]^ Colocalization of hemin inside a biofilm with eDNA was quantitated using ImageJ by correlating the positions of red and green pixels, indicating hemin and eDNA, respectively.

### Infected Wound Model in Diabetic Mice

Streptozotocin‐induced diabetic mice (male BABL/c, 17–22 g, with blood glucose levels between 10–20 mmol L^−1^ indicative of diabetes) were provided by the Chinese Academy of Medical Sciences & Peking Union Medical College. All animals were housed in the on‐site animal facility of Nankai University and experimental procedures were approved by the Institutional Animal Care and User Committee of Nankai University, Tianjin, China.

A pilot experiment was done in order to determine the dose of *S. aureus* Xen36 to create an infected wound with measurable bioluminescence intensity for at least 7 days when left untreated. For the dose‐finding pilot, animals were randomly assigned into three groups of three mice each. A wound (diameter 12 mm) was made on the back of the mice by removing the full thickness of skin using a puncher after removing the hair. Bacteria (1 × 10^8^, 5 × 10^8^, 1 × 10^9^) were added onto the wound and covered with Parafilm, fixed with surgical tape. Daily recording of bioluminescent intensities started 2 days after initiating infection using a bio‐optical imaging system IVIS Lumina II (Xenogen, California, USA) after removal of the Parafilm. Bioluminescence images were recorded with the following parameters: 45 s exposure time, medium binning, 1 F/Stop, and Open Emission Filter. Images were analyzed using ImageJ software.

Accordingly, in the final experiments, mice with a wound infected with 1 × 10^9^ staphylococci were randomly assigned in six groups of 14 mice. This assignment was made 2 days after initiating infection. Infected wounds were treated twice daily by 1) irrigation with 100 µL PBS, 2) irrigation with 100 µL 2 µg mL^−1^ ciprofloxacin dissolved in ultrapure water, 3) 100 µL G_4_‐hydrogel, 4) 100 µL GOx loaded G_4_‐hydrogel, 5) 100 µL hemin‐loaded G_4_‐hydrogel, or 6) 100 µL G_4_‐hydrogel loaded with GOx and hemin as an antimicrobial wound cover. Hydrogels were applied after removal of the previously applied hydrogel. After 4 h of first treatment, three mice of each group were randomly taken for measurement of the local glucose concentration around an infected wound. To this end, tissue was taken from within a 5 mm distance from the wound and homogenized in 1 mL ultrapure water. Glucose concentration was subsequently measured using a commercial glucose assay kit (Solarbio, Beijing, China), according to its user guide. Also, 2 mL blood was collected through the eye of the mice to test the main blood parameters of different groups after initiating treatment at day 2, day 7, and day 14.

Bioluminescent and optical imaging were performed daily, as described above. At days 2, 7, and 14, after initiating treatment, three animals in each group were sacrificed, and ≈1 g of wound tissue was taken, homogenized in 1 mL PBS, and used for plate counting. After sacrifice at day 14, wound tissue (diameter 1.5 cm) was taken and fixed with 4% formalin and embedded in paraffin. After slicing, tissues were stained with hematoxylin and eosin and examined under an optical microscope.

### Statistical Analysis

All data were expressed as means ± SD values. Differences between groups were examined for statistical significance with a one‐way ANOVA test by GraphPad Prism 8. Sample size and probability values (*p*) are indicated in the Figure captions. Differences were considered significant at *p* < 0.05.

## Conflict of Interest

H.J.B is also director‐owner of a consulting company SASA BV. The authors declare no potential conflicts of interest with respect to authorship and/or publication of this article. Opinions and assertions contained herein are those of the authors and are not construed as necessarily representing views of the funding organizations or their employer(s).

## Supporting information

Supporting Information (There is a correction in Department of Prof. Liu, so the Supporting Information should be also updated. Please check the attached new Supporting Information)

## Data Availability

The data that support the findings of this study are available from the corresponding author upon reasonable request.
