# Supplementary material for: A Guanosine‐Quadruplex Hydrogel as Cascade Reaction Container Consuming Endogenous Glucose for Infected Wound Treatment—A Study in Diabetic Mice
Source: Adv Sci (Weinh). 2022 Jan 22;9(7):2103485. doi: 10.1002/advs.202103485 (PMC8895150; doi:10.1002/advs.202103485)
Supplement: Supplementary file 1 — Supporting Information (There is a correction in Department of Prof. Liu, so the Supporting Information should be also updated. Please check the attached new Supporting Information) [file ADVS-9-2103485-s001.pdf]

ADVANCED SCIENCE
Open Access

Supporting Information

for *Adv. Sci.*, DOI: 10.1002/advs.202103485

A G-quadruplex Hydrogel as Cascade Reaction Container Consuming Endogenous Glucose for Infected Wound Treatment – a Study in Diabetic Mice

Yuanfeng Li, Linzhu Su, Yongxin Zhang, Yong Liu, Fan Huang*, Yijin Ren, Yingli An, Linqi Shi*, Henny C. van der Mei*, Henk J. Busscher*

Supporting Information

A G-quadruplex Hydrogel as Cascade Reaction Container Consuming Endogenous Glucose for Infected Wound Treatment – a Study in Diabetic Mice

Yuanfeng Li[1,2], Linzhu Su[1,2], Yongxin Zhang[1], Yong Liu[3], Fan Huang[4]*, Yijin Ren[5], Yingli An[1], Linqi Shi[1]*, Henny C. van der Mei[2]*, Henk J. Busscher[2]*

Y. Li, L. Su, Y. Zhang, Y. An, Prof. L. Shi
[1] State Key Laboratory of Medicinal Chemical Biology, Key Laboratory of Functional Polymer Materials, Ministry of Education, Institute of Polymer Chemistry, College of Chemistry, Nankai University, 94 Weijin Road, Tianjin 300071, P. R. China
E-mail: shilinqi@nankai.edu.cn

Y. Li, L. Su, Prof. H.C. van der Mei, Prof. H.J. Busscher
[2] University of Groningen and University Medical Center Groningen, Department of Biomedical Engineering, Antonius Deusinglaan 1, 9713 AV Groningen, The Netherlands.
E-mail: h.c.van.der.mei@umcg.nl

Prof. Y. Liu
[3] Wenzhou Institute, University of Chinese Academy of Sciences, Oujiang Laboratory (Zhejiang Lab for Regenerative Medicine, Vision and Brain Health), 1 Jinlian Road, Longwan District, Wenzhou 325001, P. R. China.
E-mail: y.liu@ucas.ac.cn

Prof. F. Huang
[4] Tianjin Key Laboratory of Radiation Medicine and Molecular Nuclear Medicine, Institute of Radiation Medicine, Chinese Academy of Medical Science & Peking Union Medical College, 238 Baiti Road Tianjin 300192, P. R. China.
E-mail: huangfan@irm-cams.ac.cn

Prof. Y. Ren
[5] University of Groningen and University Medical Center Groningen, Department of Orthodontics, Hanzeplein 1, 9700 RB Groningen, The Netherlands

SUPPORTING FIGURES

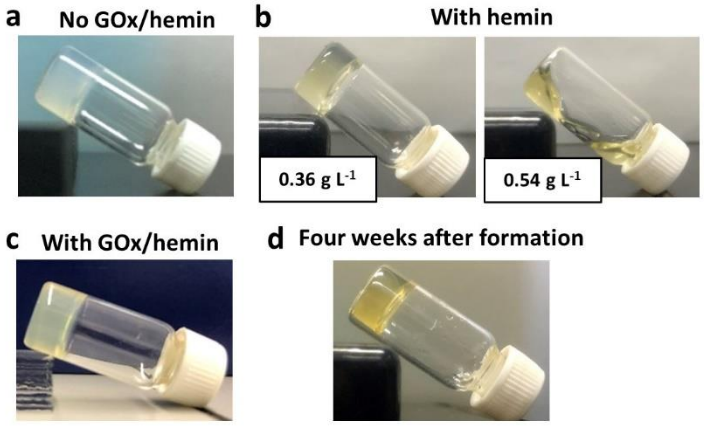

Figure S1. Gelation of G_4-hydrogels, self-assembled from a mixture of guanosine, 2-formylphenylboronic acid (2-FPBA), putrescine and KCl at a molar ratio of 1 : 1 : 0.5 : 0.25. Photographs were taken after 2 h after gel formation unless stated otherwise. **a)** G_4-hydrogel in absence of GOx/hemin loading. **b)** Hemin loading only. **c)** GOx/hemin loading. **d)** GOx/hemin loaded G_4-hydrogel four weeks after formation. Note yellowish discoloration.

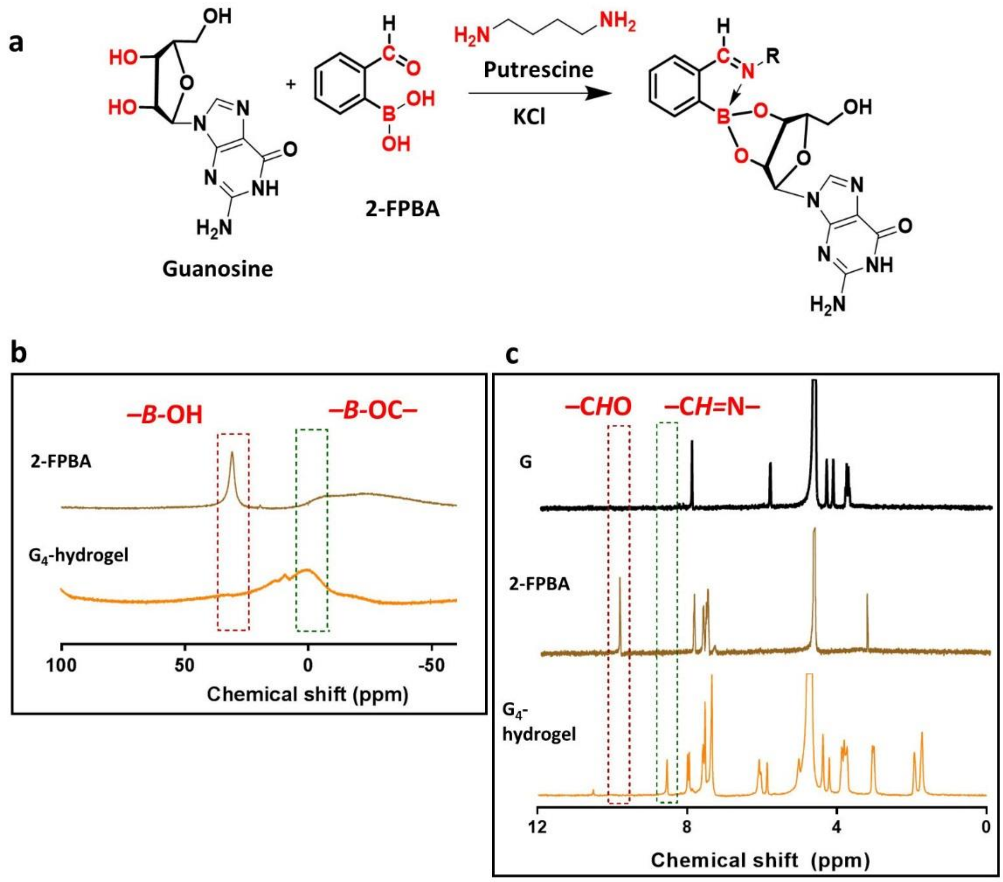

Figure S2. Reaction scheme of G_4-quadruplex structure and putrescine-mediated formation of iminoboronate bonds. Guanosine-quadruplex(G_4)-hydrogels were self-assembled from a mixture of guanosine, 2-formylphenylboronic acid (2-FPBA), putrescine (Put) and KCl at a molar ratio of 1 : 1 : 0.5 : 0.25 in D_2O. **a)** Schematic of iminoboronate bond formation. **b)** ^{11}B NMR spectra of 2-FPBA after coupling of guanosine. **c)** ^{1}H NMR spectra of putrescine-mediated 2-FPBA G_4-hydrogels. All NMR spectra of G_4-quadruplex hydrogels and 2-FPBA are taken in D_2O.

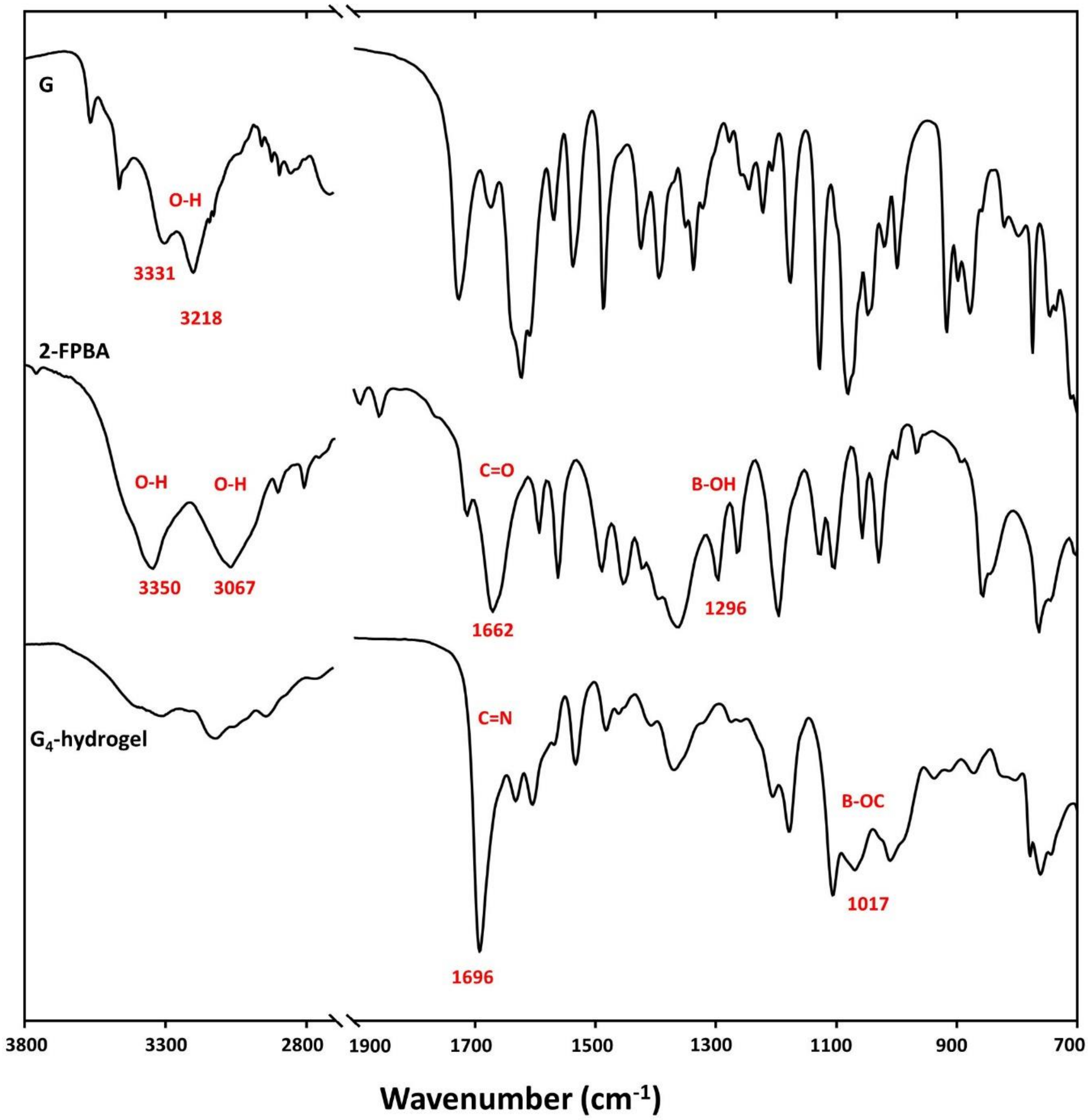

Figure S3. FTIR spectra of guanosine (G), 2-formylphenylboronic acid (2-FPBA) and putrescine-mediated G_4-hydrogels.

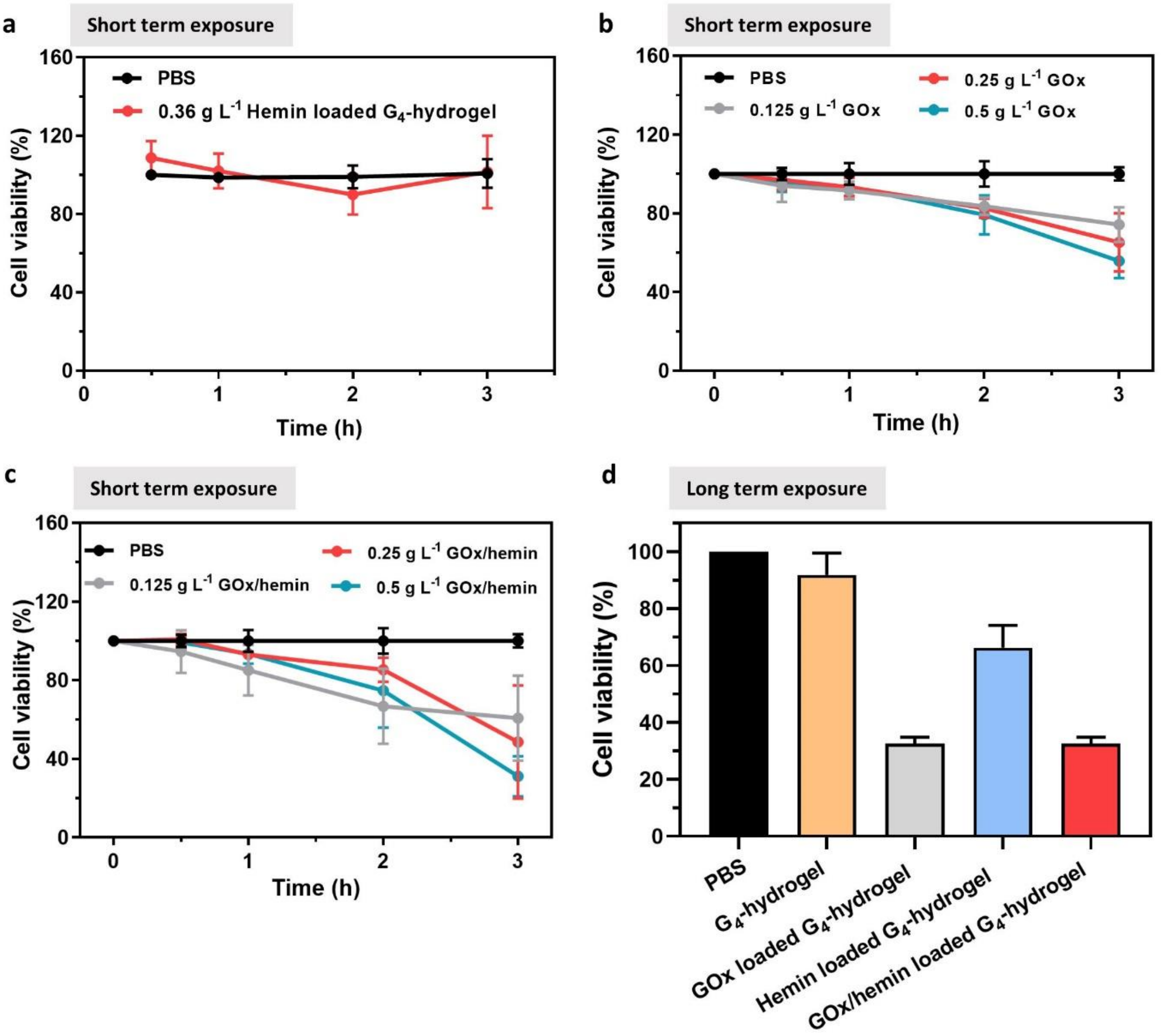

Figure S4. Viability of human skin fibroblasts exposed to differently loaded G_4-hydrogels. Hemin and GOx only loading were done at 0.36 g L^{-1} and 0.25 g L^{-1}, respectively. Viability of human skin fibroblasts is expressed as a percentage metabolic activity, as a function of time in high glucose DMEM (4.5 g L^{-1} glucose) medium in presence of hydrogels. **a)** Cell viability upon short term exposure (3 h) to G_4-hydrogels loaded with hemin. **b)** Same as panel a, for GOx loading only. **c)** Same as panel a, for loading with GOx and hemin. **d)** Cell viability upon long term exposure (24 h) to unloaded and differently loaded G_4-hydrogels. Data represent means ± standard deviations over triplicate runs with different fibroblast cultures and separately prepared hydrogels.

Method: Viability of human skin fibroblasts in mono-cultures was studied through an MTT (3-(4,5-dimethylthiazoly-2)-2,5-diphenyltetrazolium bromide) assay. Human skin fibroblasts (CCD-1112Sk) were cultured in a 5% CO_2 atmosphere at 37°C in DMEM medium containing 4.5 g L^{-1} glucose and 10% (v/v) fetal bovine serum and harvested by trypsinization at 80%

confluency. Fibroblasts suspended in high glucose (4.5 g L^{-1}) DMEM were seeded into gelatin-coated 96-wells plates (1 × 10^4 cells per well) and incubated in a 5% CO_2 atmosphere at 37°C for 12 h. After removal of the growth medium, hydrogels were added to cover the cells and fresh, high glucose DMEM was added. After exposure for different time intervals up to 3 h (short term exposure) or 24 h (long term exposure) at 37°C, growth medium was removed and the cells were washed three times with PBS, after which 100 μL MTT (Sigma-Aldrich, 1 mg mL^{-1}) in DMEM was added. After 4 h, the supernatant was removed and 100 μL isopropanol was added to dissolve formazan and absorbance was measured at 560 nm on a Fluostar® microplate reader (Ortenberg, Germany).

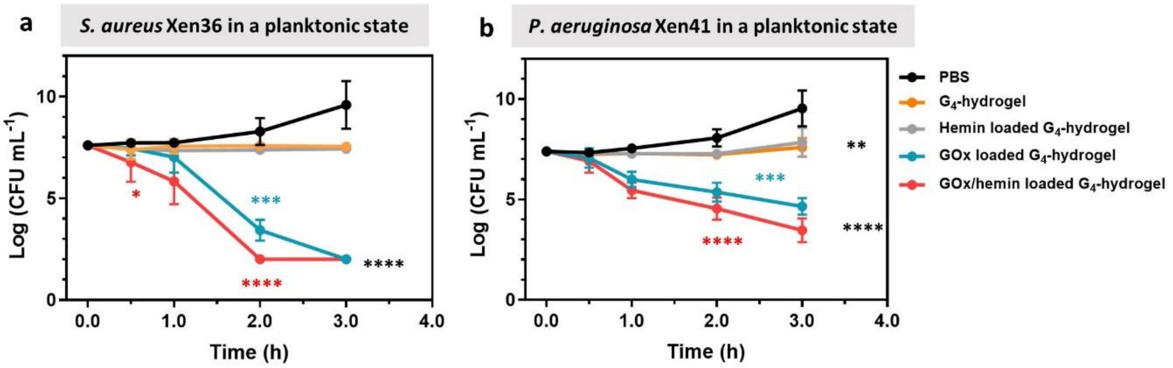

Figure S5. The number of planktonic *S. aureus* Xen36 or *P. aeruginosa* Xen41 in suspension as a function of time in tryptone soy broth supplemented with glucose at a concentration of 5 g L^{-1} and in presence of differently loaded G_4-hydrogels. Hemin and GOx only loading were done at 0.36 g L^{-1} and 0.25 g L^{-1}, respectively. Data represent means ± standard deviations over triplicate runs with different bacterial cultures and separately prepared hydrogels. Asterisks indicate statistical significance at *$p < 0.05$, ** $p < 0.01$, *** $p < 0.001$ and **** $p < 0.0001$ (one-way ANOVA test) between differences with respect to exposure to PBS in absence of hydrogels.

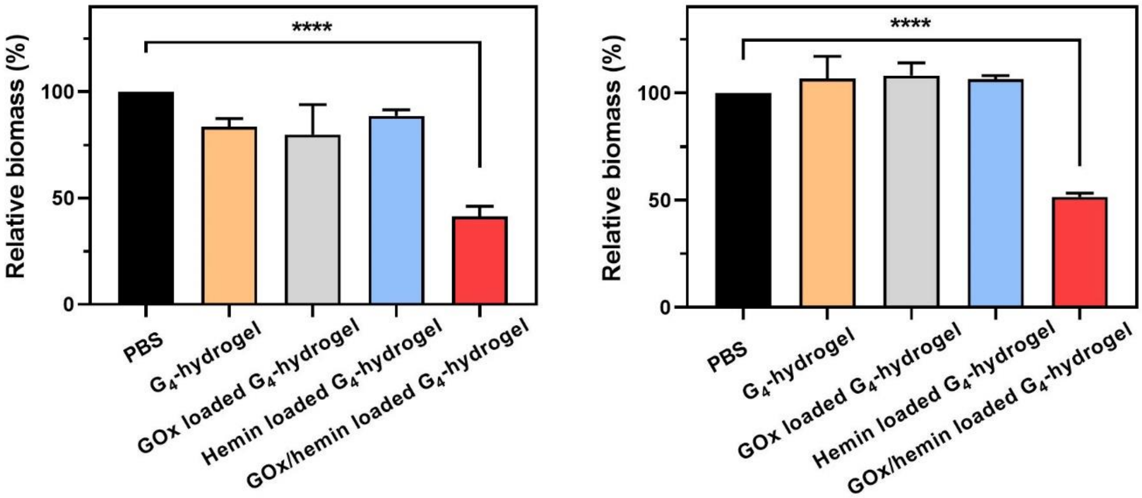

Figure S6. Relative biomass of *S. aureus* Xen36 or *P. aeruginosa* Xen41 biofilms after exposure to differently loaded G_4-hydrogels, setting the biomass in PBS in absence of hydrogel exposure at 100%. Hemin and GOx only loading were done at 0.36 g L^{-1} and 0.25 g L^{-1}, respectively. Data were expressed as means ± standard deviations over triplicate experiments with independently grown biofilms and separately prepared hydrogels.

Method: 48 h biofilms were grown and exposed to PBS, unloaded G_4-hydrogels or hydrogels loaded with only GOx or hemin or GOx/hemin in tryptone soy broth supplemented with glucose (5 g L^{-1}) for 8 h. Next, biofilms were stained with crystal violet (0.5%, w/v) for 20 min. Crystal violet solution was removed and biofilms were gently washed with PBS for three times. 1 mL of 33% acetic acid was added to resuspend the stained biofilm for 15 min. The absorbance of each sample was recorded by a microplate reader (Spark, TECAN, Switzerland) at 595 nm.

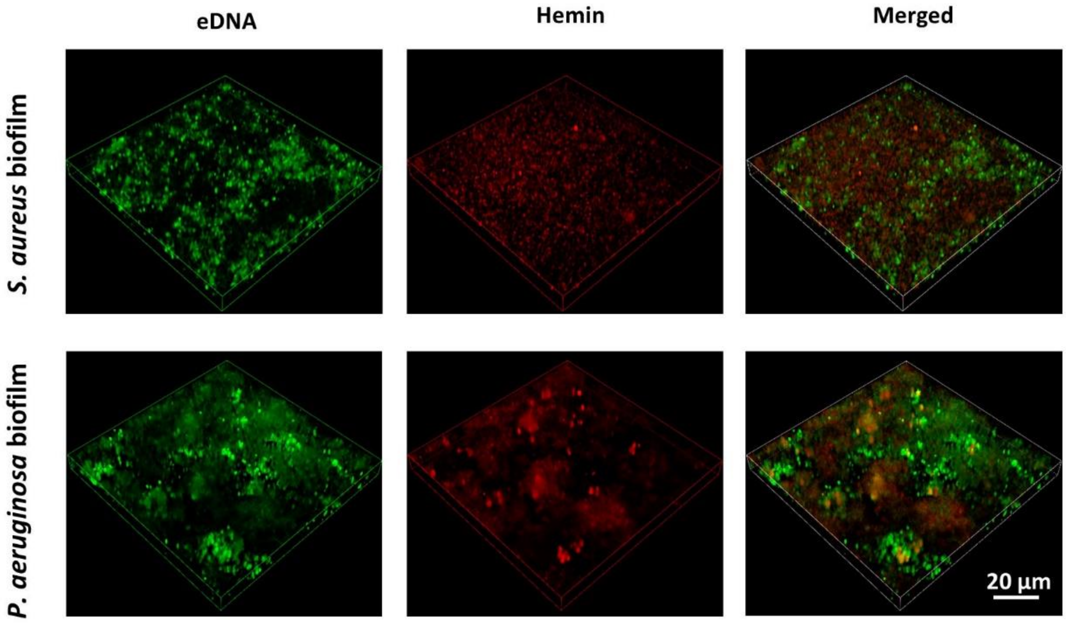

Figure S7. Penetration and accumulation of hemin and presence of eDNA G-quadruplexes in the EPS matrix of 48 h old *S. aureus* ATCC 12600 and *P. aeruginosa* PAO1 biofilms. eDNA G-quadruplex nucleic acid structures in the EPS matrix of biofilms were visualized by binding of anti-DNA G-quadruplex structures antibody and green-fluorescently-labelled goat anti-mouse IgG. Hemin carries red fluorescence of its own.

Method: 48 h biofilms were grown and covered with GOx/hemin loaded G_4-hydrogels for 4 h. Next, biofilms were fixed in 4% paraformaldehyde for 15 min at room temperature, washed three times with PBS and exposed to 5% fetal bovine serum for 1 h, followed by treatment of G-quadruplex DNA specific primary antibody (Sigma-Aldrich, CAT#MABE1126) for 1 h, and washed three times with PBS. Subsequently, green-fluorescently-labelled (Alexa Fluor 488) goat anti-mouse IgG (Solarbio, CAT#SF131) was added for 1 h, followed by three times washing with PBS. eDNA G-quadruplex nucleic acid structures and penetration and accumulation of hemin in the biofilms were visualized using CLSM.[1]

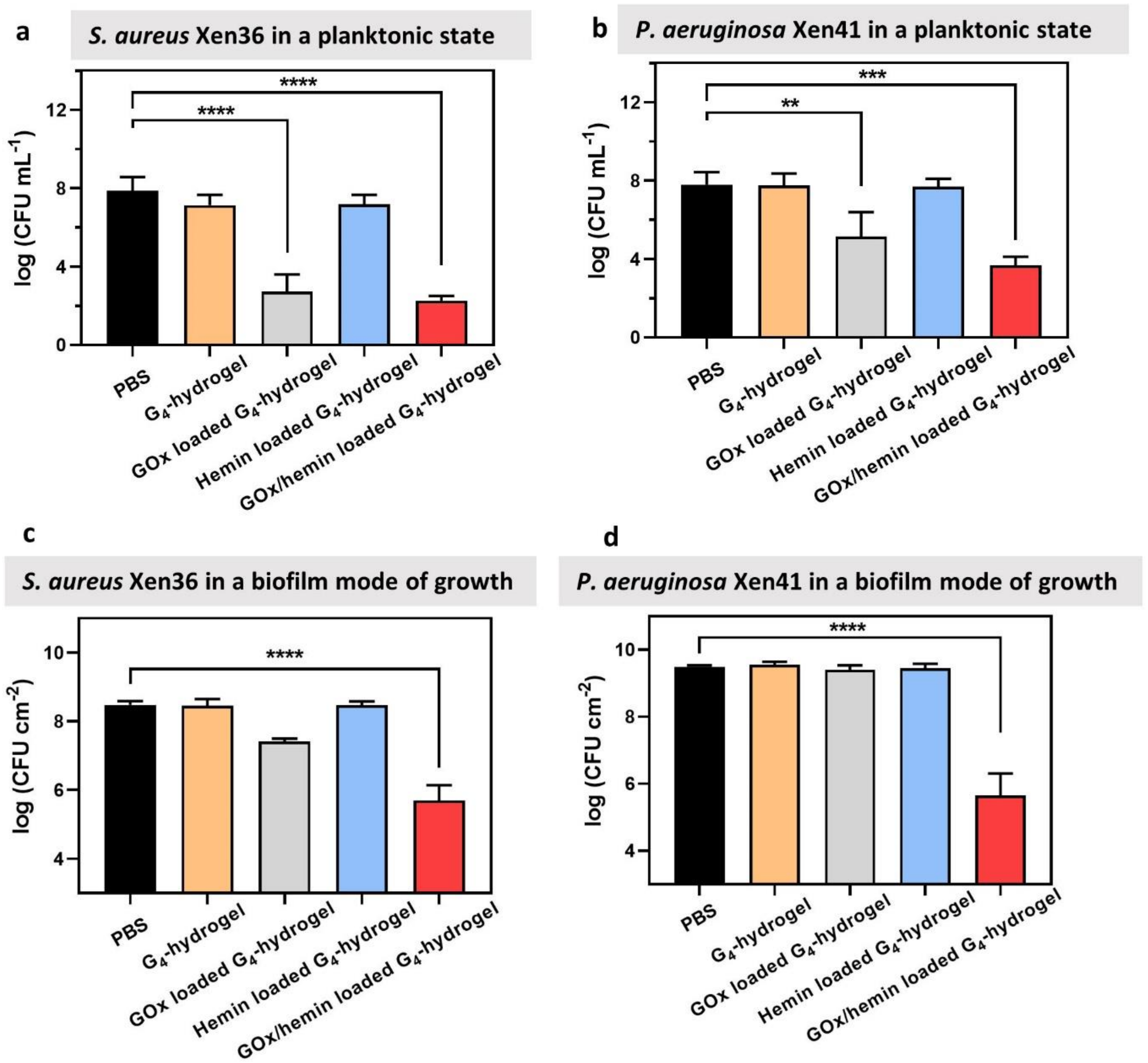

Figure S8. Influence of hydrogel storage for four weeks on bacterial killing by differently loaded G_4-hydrogels reactions. Hemin and GOx only loading were done at 0.36 g L^{-1} and 0.25 g L^{-1}, respectively. **a)** The number of planktonic *S. aureus* Xen36 suspended in tryptone soy broth supplemented with glucose (5 g L^{-1}) after 3 h growth in presence of stored, differently loaded G_4-hydrogels. Data were expressed as means ± standard deviations over triplicate experiments with independently grown biofilms and separately prepared hydrogels. **b)** Same as panel a, now for *P. aeruginosa* Xen41. **c)** The number of CFU cm^{-2} after 8 h exposure of *S. aureus* Xen36 biofilms to stored, differently loaded G_4-hydrogels. Data were expressed as means ± standard deviations over triplicate experiments with independently grown biofilms and separately prepared hydrogels. **d)** Same as panel c, now for *P. aeruginosa* Xen41. Asterisks indicate statistical significance at ** $p < 0.01$, *** $p < 0.001$ and **** $p < 0.0001$ (one-way ANOVA test) between differences with respect to exposure to PBS in absence of hydrogels.

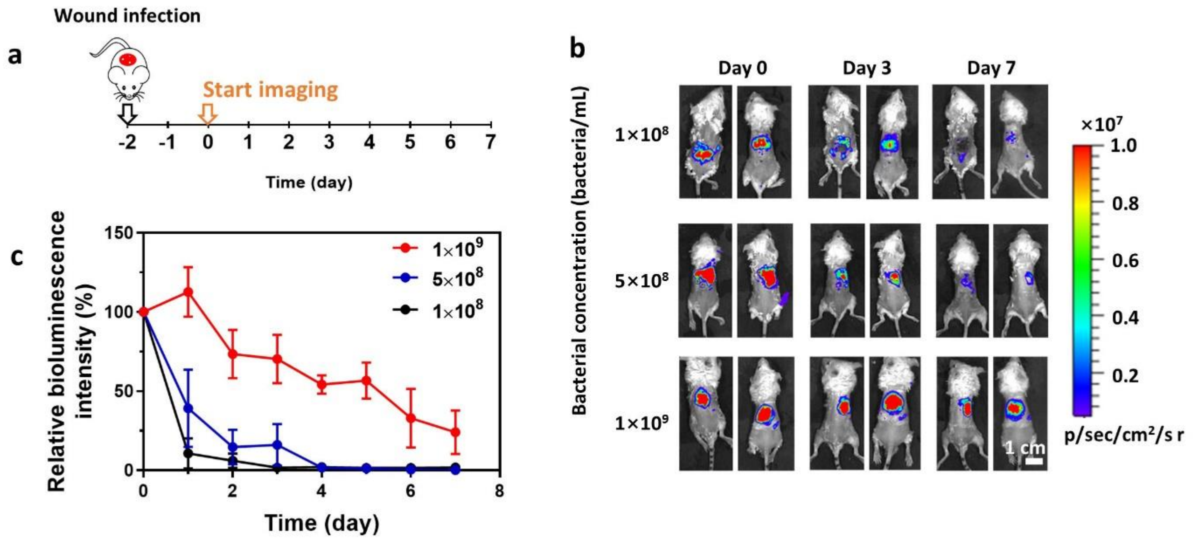

Figure S9. Dose-finding pilot for creating an infected wound in diabetic mice. **a)** Experimental scheme: *S. aureus* Xen36 was added onto an open wound at different doses two days prior to starting bioluminescence imaging at day 0 to monitor the course of infection**. b)** Bioluminescence images of mice infected with different staphylococcal doses, taken at day 3 and 7 after initiating imaging in the same mouse (images are presented of two mice per group. **c)** Percentage bioluminescence intensity arising from the infection site of mice infected with different staphylococcal doses as a function of time after initiating imaging. Bioluminescence intensity at day 0 was set at 100%. All quantitative data are presented as means ± standard deviations over three mice in each group.

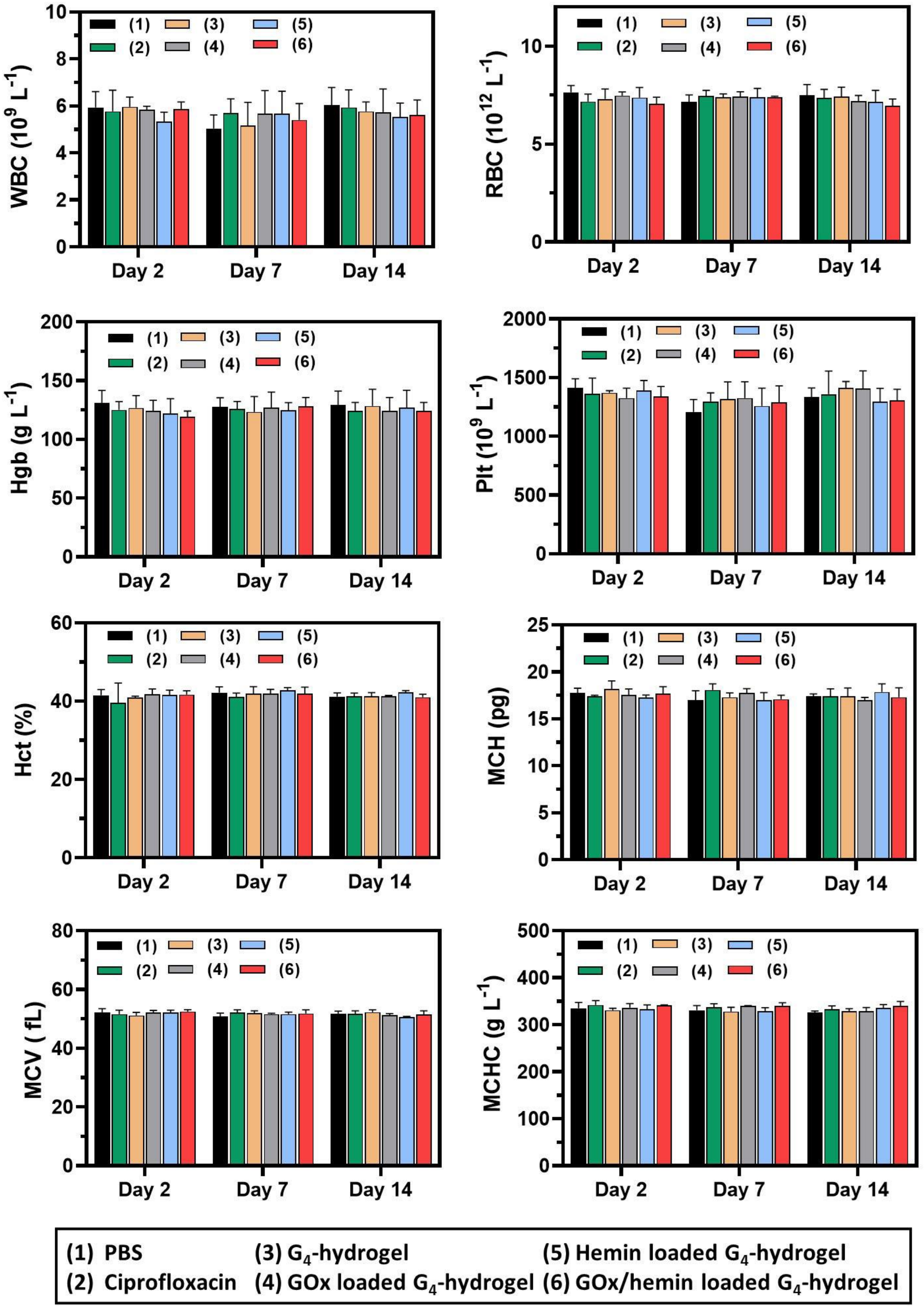

Figure S10. Routine blood parameters of diabetic mice, with wounds infected with *S. aureus* Xen36 (1×10^9 bacteria per wound site) at different days after initiating treatment. Treatment involved irrigation of the wound with PBS (100 μL) or a ciprofloxacin solution (100 μL, 2 μg mL^{-1}) or coverage of the wound with 100 μL of differently loaded G_4-hydrogels. Hemin and GOx only loading were done at 0.36 g L^{-1} and 0.25 g L^{-1}, respectively. Irrigation was repeated and hydrogels replaced by fresh ones every 12 h. Parameters included: white blood cell (WBC,

reference value 0.8-6.8 10^9 L^{-1}), red blood cell (RBC, reference value 6.36-9.42 10^{12} L^{-1}), hemoglobin (Hgb, reference value 110-143 g L^{-1}), platelet (Plt, reference value 450-1590 10^9 L^{-1}), hematocrit (Hct, reference value 34.6-44.6%), mean corpuscular hemoglobin (MCH, reference value 15.8-19.0 pg), mean corpuscular volume (MCV, reference value 48.2-58.3 10^{-15} L) and mean corpuscular hemoglobin concentration (MCHC, 302-353 g L^{-1}). Error bars denote SD over three mice in each group.

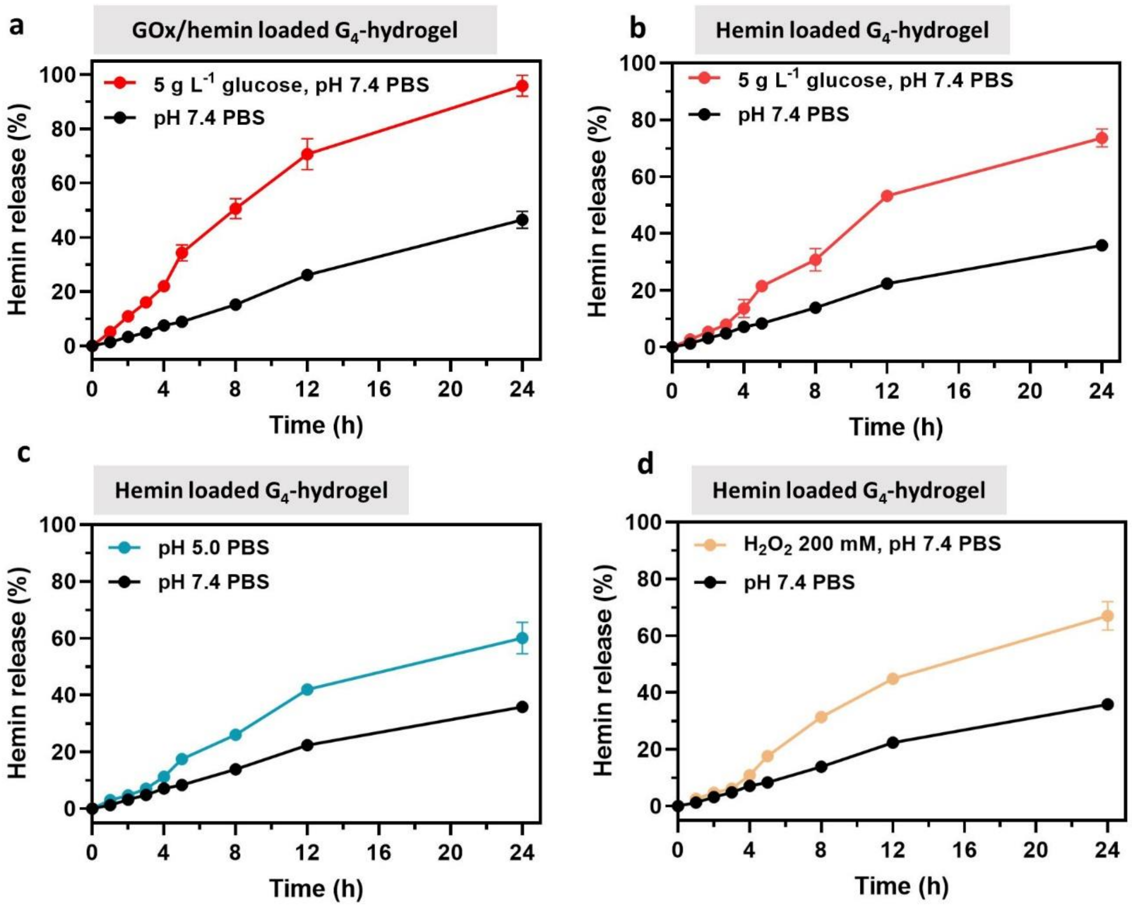

Figure S11. Factors stimulating hemin release from differently loaded G_4-hydrogels. **a)** Hemin release from GOx/hemin loaded G_4-hydrogels. **b)** Hemin release from hemin only loaded G_4-hydrogel (0.36 g L^{-1}) as a function of time in absence and presence of 5 g L^{-1} glucose (PBS, pH 7.4. **c)** Hemin release from hemin only loaded G_4-hydrogels as a function of time at pH 5.0 and 7.4 (PBS). **d)** Hemin release from hemin only loaded G_4-hydrogels as a function of time in absence and presence of H_2O_2 (200 mM H_2O_2) in PBS (pH 7.4). Data represent means ± standard deviations over triplicate runs with different preparation of hydrogels.

Method: 1 mL hemin loaded or GOx/hemin loaded G_4-hydrogel was prepared in 12-well plates. Next, 3 mL different hemin-release stimulating factors, including glucose, acidic conditions or H_2O_2 presence were imposed after which fluorescence emission of supernatant at 630 nm with 410 nm excitation were recorded at different time as a measure of hemin release. Hemin release was expressed with respect to the total amount of hemin in a hydrogel,

determined upon measuring the fluorescence emission of 1 mL of a hydrogel after boiling until a clear solution resulted and after dilution in 3 mL PBS (pH 7.4).

Table S1. Minimal inhibitory (MIC) and minimal bactericidal (MBC) concentrations (µg mL^{-1}) of five antibiotics against the four bacterial strains included in this study. *S. aureus* Xen36 and *P. aeruginosa* Xen41 are laboratory strains, while *S. aureus* df1 and *P. aeruginosa* df2 are clinical isolates from patients with diabetic foot ulcers. "R" indicates resistance of a strain against the antibiotic, while "S" indicates susceptibility. "I" indicates intermediate resistance.[2,3]

**Antibiotic**	*S. aureus* Xen36			*S. aureus* df1			*P. aeruginosa* Xen41			*P. aeruginosa* df2		
	**MIC**	**MBC**	**R/S**	**MIC**	**MBC**	**R/S**	**MIC**	**MBC**	**R/S**	**MIC**	**MBC**	**R/S**
**Vancomycin**	0.5	1	S	1	1	S	>256	>256	R	>256	>256	R
**Piperacillin**	8	32	R	8	16	R	2	2	S	4	8	I
**Gentamicin**	2	2	S	4	4	I	1	8	S	1	1	S
**Clindamycin**	<0.25	4	S	<0.25	4	S	>256	>256	R	>256	>256	R
**Ciprofloxacin**	2	2	S	<0.25	8	S	<0.25	1	S	<0.25	0.5	S

Method: The MIC and MBC of the four bacterial strains were determined for vancomycin, piperacillin, gentamicin, clindamycin and ciprofloxacin. To measure the MIC and MBC, antibiotics in TSB were used in an antibiotic range of 256 to 0.5 µg mL^{-1} in 96 well-plates and mixed with 100 µL of a bacterial suspension (2×10^5 bacteria per mL) in TSB. Wells were incubated at 37°C for 24 h and the density was scored visually. The MIC was taken as the lowest antibiotic concentration at which no turbidity of the suspension was observed. 10 µL of each clear suspension was plated onto TSB agar plates, followed by incubation at 37°C for 24 h. The MBC values were taken as the lowest antibiotic concentration at which visual bacterial growth was absent on the agar plates.

REFERENCES

[1] T. Seviour, F. R. Winnerdy, L. L. Wong, X. Shi, S. Mugunthan, G. S. Kohli, H. M. Shewan, J. R. Stokes, S. A. Rice, A. T. Phan, S. Kjelleberg, bioRxiv **2019**.

DOI:10.1101/527267. https://www.biorxiv.org/content/10.1101/527267v1, accessed Dec 2020.

[2] T. J. Foster, *FEMS Microbiol. Rev.* **2017**, *41*, 430.

[3] C. J. Henwood, D. M. Livermore, D. James, M. Warner, P. S. Group, J. *Antimicrob. Chemother.* **2001**, *47*, 789.
